# Case report: Transvaginal single-port extraperitoneal laparoscopic sacrospinous ligament fixation for apical prolapse: A single-center case series

**DOI:** 10.3389/fsurg.2023.1066622

**Published:** 2023-03-29

**Authors:** Ye Liu, Chao Wang, Xianjing Wang, Rongrong Yan, Lei Chu, Xinliang Chen

**Affiliations:** ^1^ The International Peace Maternity and Child Health Hospital, School of Medicine, Shanghai Jiao Tong University, Shanghai, China; ^2^ Shanghai Key Laboratory of Embryo Original Diseases, Shanghai, China

**Keywords:** sacrospinous ligament fixation, transvaginal, single-port laparoscopy, POP, video

## Abstract

**Background:**

Sacrospinous ligament fixation (SSLF) is a minimally invasive and effective procedure for the treatment of apical prolapse. Because intraoperative exposure of the sacrospinous ligament is difficult, SSLF is difficult. The aim of our article is to determine the safety and feasibility of single-port extraperitoneal laparoscopic SSLF for apical prolapse.

**Methods:**

This single-center, single-surgeon case series study included 9 patients with pelvic organ prolapse quantification (POP-Q) III or IV apical prolapse who underwent single-port laparoscopic SSLF. Additionally, transobturator tension-free vaginal tap (TVT-O) was performed in 2 patients, and anterior pelvic mesh reconstruction was performed in 1 patient.

**Results:**

The operative time ranged from 75 to 105 (mean, 88.9 ± 10.2) min, and blood loss ranged from 25 to 100 (mean, 43.3 ± 22.6) ml. No serious operative complications, blood transfusions, visceral injuries, or postoperative gluteal pain were reported for these patients. After 2–4 months of follow-up, no recurrence of POP, gluteal pain, urinary retention/incontinence, or other complications was observed.

**Conclusion:**

Transvaginal single-port SSLF is a safe, effective, and easy-to-master operation for apical prolapse.

## Background

Pelvic organ prolapse (POP) is a highly prevalent disease that has a serious impact on quality of life (1). As the aging population grows, the prevalence of POP in elderly women is gradually increasing, i.e., according to projections, the number of patients with POP in the United States will increase from 3.3 million to 4.9 million in the next 40 years (2). Currently, more than 220,000 patients require surgery to treat POP each year (3). Sacrospinous ligament fixation (SSLF) was first proposed by Sederl in Germany in 1958 to resolve the fixation of the vaginal tip to the sacrotuberous ligament (4). In 1967, Richter improved this approach by suturing the apex of the vagina to the sacrospinous ligament (SSL). The operation has benefited a large number of patients suffering from prolapse. According to previous studies, the subjective success rate of SSLF is 84%–99%, and the objective success rate is 67%–93% (5). However, the SSL is located deep in the pelvic cavity, making it difficult to expose during transvaginal surgery. It is also surrounded by several important nerves and blood vessels. If the suture cannot be placed in the correct position during SSLF, the peripheral blood vessels and nerves may be injured, causing complications such as pain, hemorrhage, infection, and fistula (6). Gluteal pain is a common complication of SSLF surgery, with a reported postoperative incidence ranging from 6.1% to 84%. It is usually caused by a neurologic injury sustained when the SSL is sutured (7–11), and some patients require medication or local anesthesia to treat the pain (11–14). According to a previous study, blood transfusion was required during this operation in 4.3% of patients due to intraoperative vascular injury (15). This article proposed a new technique for SSLF using transvaginal single-port extraperitoneal laparoscopy for severe POP. Compared with traditional SSLF, this new operation is performed under direct vision and enables the operator to expose the SSL, thus avoiding injuries to the blood vessels and nerves. Because this new technique is easy to learn and master, its application in SSLF should be further promoted in clinical practice.

## Methods

This prospective single-center, single-surgeon case series study included 9 patients with pelvic organ prolapse quantification (POP-Q) stage III or IV apical prolapse between November 2021 and March 2022. All the patients were between 35 and 85 years of age, and had uterine or vaginal stump prolapse POP-Q scores of III or higher, indicating a significant effect on daily life. None of the included patients were in the acute infection phase, had any combination of serious liver, kidney, cardiac, hematological or neurological diseases, diabetes mellitus, malignancy or psychiatric disorders, or history of using drugs that would affect coagulation function, etc. Preoperative POP-Q staging and incontinence provocation tests were performed for all the enrolled patients. After the patient provided informed consent, transvaginal single-port extraperitoneal laparoscopic SSLF was performed by an experienced urogynecologist who had performed more than 500 transvaginal SSLF procedures. Patients were assessed daily until discharge to observe the occurrence of postoperative complications, and outpatient follow-up was performed 1 month after the operation, after which the patients were followed up monthly by telephone. Information on surgery-related complications, especially gluteal pain and POP recurrence, was collected.

### Surgical technique

**Figure 1:**
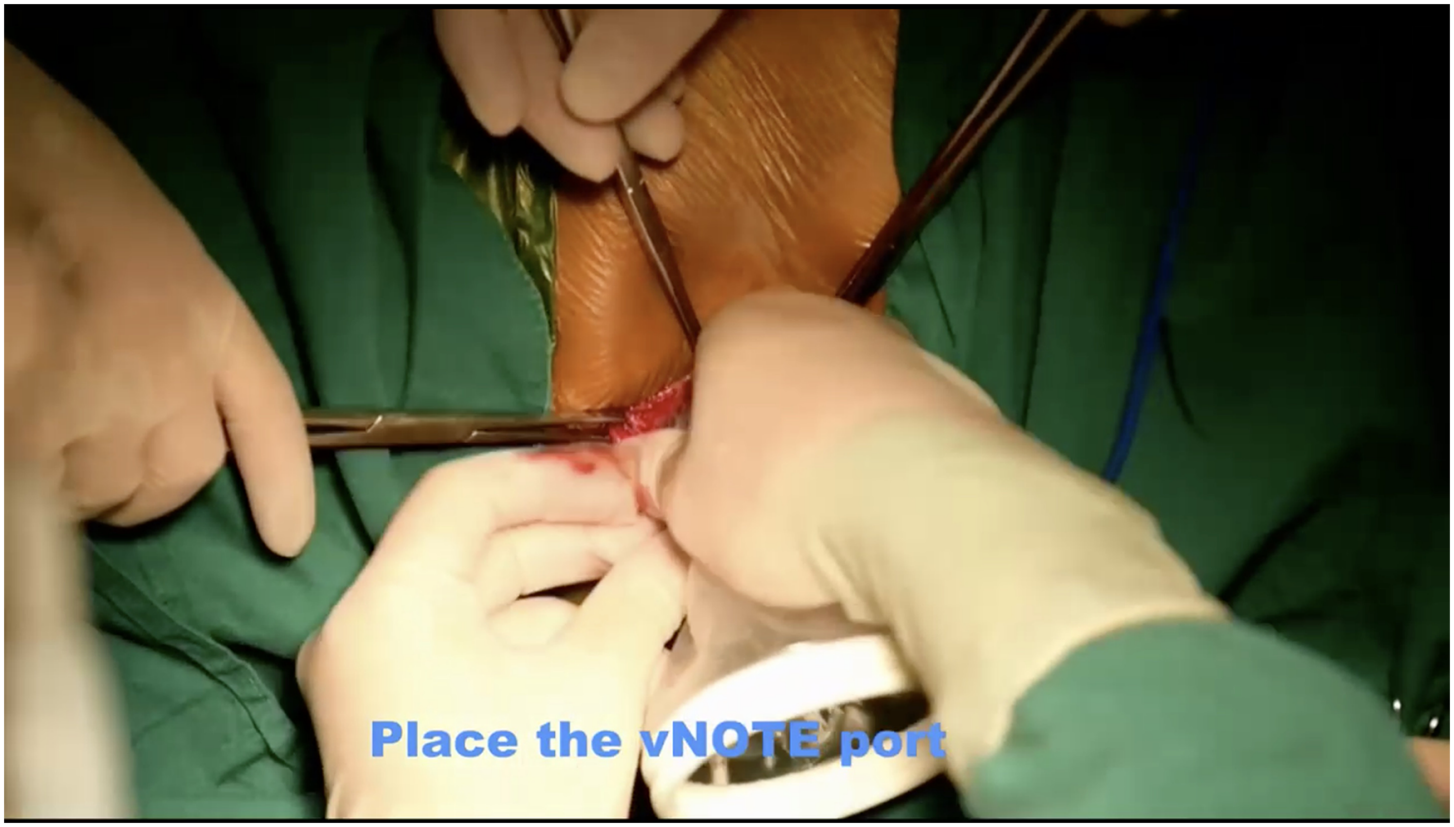
Placement of single-port devices. The laparoscopic incision protector was placed inside the posterior vaginal wall incision and made contact with the tissues..

**Figure 2:**
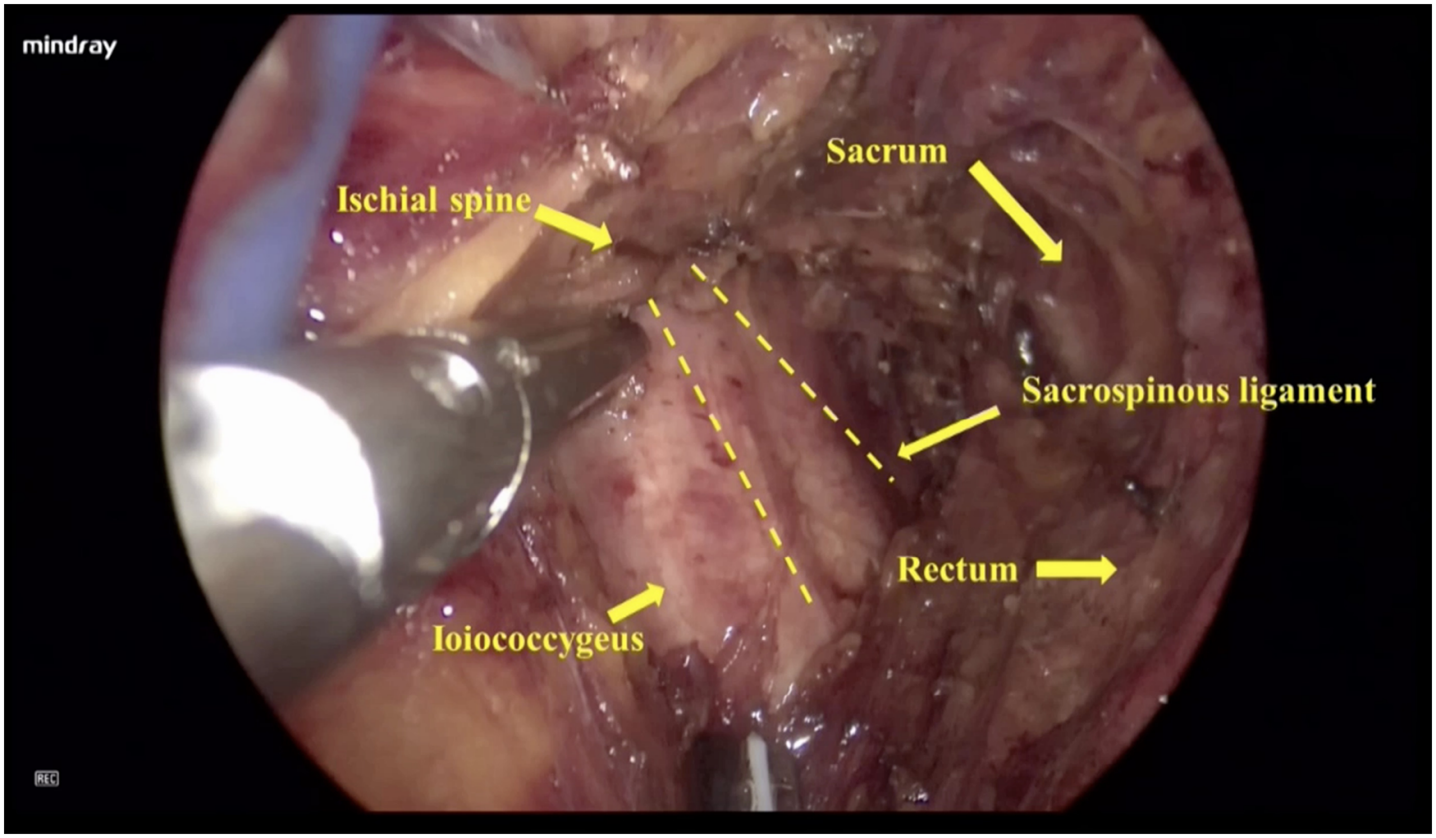
Sacrospinous ligament exposure. An ultrasonic scalpel was used to laparoscopically separate the loose tissues next to the rectum until the sacrospinous ligament, the adjacent piriformis, and iliococcygeal muscles were fully exposed.

**Figure 3:**
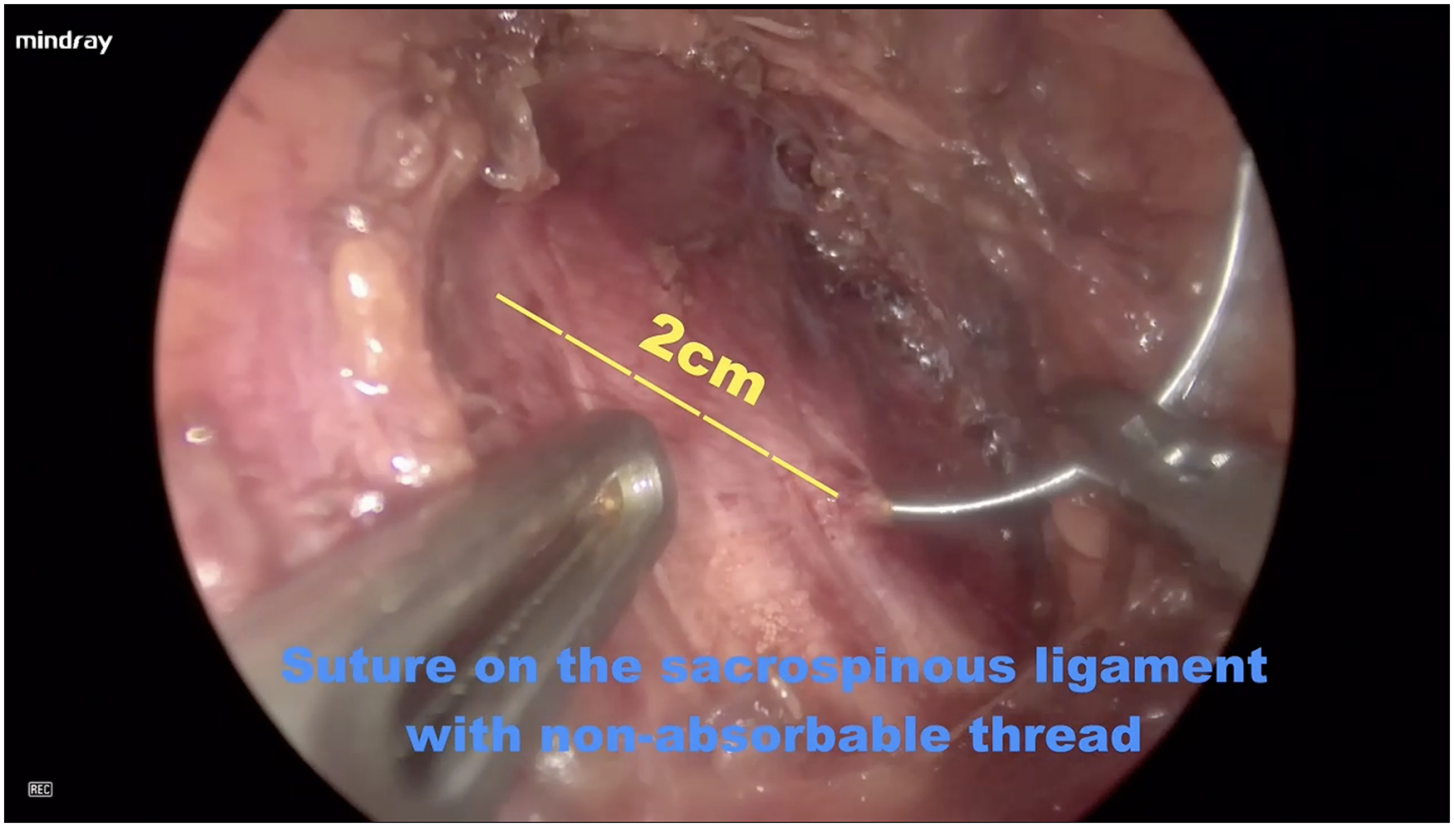
Sacrospinous ligament suturing. The right sacrospinous ligament was sutured with a nonabsorbable suture using a circular needle (O1/2 10 × 20) approximately 2 cm from the ischial spine at a depth of ≤0.3 cm.

The transvaginal single-port laparoscopic SSLF procedure was performed as follows (Supplementary Material):

Step 1: Posterior colpotomy. The patients were administered general anesthesia and placed in the lithotomy position. After endotracheal intubation, 50 ml normal saline was injected between the posterior vaginal wall and the rectum to form a water cushion. Then, a 4 cm longitudinal incision was made from the hymen ring to the middle of the posterior vaginal wall.
Step 2: Pararectal space dissection. Blunt and sharp dissection of the right pararectal space was performed until the ischial spine and SSL were identified.
Step 3: Placement of single-port devices. The laparoscopic incision protector was placed inside the posterior vaginal wall incision and made contact with the tissues (Figure 1). The single port device (HK-FDDC-4FGD, HTKD Med) was established. Subsequent low-flow CO2 insufflation is required to maintain the operating space and view, usually at 2/3 of the normal laparoscopic flow rate.
Step 4: Sacrospinous ligament exposure. A 30° 10-mm laparoscope was placed into the pararectal space. An ultrasonic scalpel was used to laparoscopically separate the loose tissues next to the rectum until the sacrospinous ligament, the adjacent piriformis, and iliococcygeal muscles were fully exposed (Figure 2). When necessary, the assistant placed a finger in the rectum to locate the ischial spine.
Step 5: Sacrospinous ligament suturing. The right sacrospinous ligament was sutured with a nonabsorbable suture using a circular needle (O1/2 10 × 20) approximately 2 cm from the ischial spine at a depth of ≤0.3 cm (Figure 3). The coccygeal muscle was identified and avoided during suturing; the suture was kept in reserve.
Step 6: Anterior colpotomy. Fifty milliliters of normal saline was injected between the bladder and the anterior vaginal wall to form a water cushion. A 4 cm longitudinal incision was made from the vaginal vault to the anterior vaginal wall. The bladder-cervical space was separated by scissors, and the bladder was pushed away.
Step 7: Cervix suturing. The posterior vaginal incision was prolonged to the posterior fornix. Two-thirds of the right wall of the cervical canal was punctured with the previously indwelling nonabsorbable suture.
Step 8: Vaginal wall closure. The incisions made in the anterior vaginal wall and the upper part of the posterior vaginal wall were closed with 2–0 Vicryl sutures. When necessary, a transobturator tension-free vaginal tap (TVT-O) was performed.
Step 9: Tightening the suture. The nonabsorbable suture was tightened, and the cervix was pushed up until it reached the level of the ischial spine. The lower part of the posterior vaginal wall incision was closed with a 2–0 Vicryl suture.
Step 10: Checking and storing. A urethral catheter was placed overnight. A vaginal disinfectant was applied, and gauze was packed in the vagina for 24 h.

## Results

A total of 9 patients with ages ranging from 62 to 76 (mean, 70.8 ± 4.8) years were included. Gravidity ranged from 2 to 6 (mean, 3.8 ± 1.6), and parity ranged from 1 to 4 (mean, 4.0 ± 2.3). None of the patients had a history of hysterectomy; 4 patients were diagnosed with a POP-Q stage 4 apical prolapse and 5 patients with a POP-Q stage 3 apical prolapse; 3 patients were complicated with a POP-Q stage 3 anterior vaginal wall prolapse, and 2 patients were complicated with stress urinary incontinence (SUI).

All operations were successfully completed. In addition to apical repair, concomitant TVT-O (*n* = 2) and anterior pelvic mesh reconstruction (*n* = 1) were performed when needed. The operative time ranged from 75 to 105 (mean, 88.9 ± 10.2) min, and blood loss ranged from 25 to 100 (mean, 43.3 ± 22.6) ml. There were no serious operative complications, perioperative blood transfusions, visceral injuries, or postoperative gluteal pain. All patients were discharged 2 days after the operation. After 2–4 months of follow-up, there was no recurrence of POP, gluteal pain, urinary retention/incontinence, or any other complications.

## Discussion

There are numerous surgical options available for the treatment of uterine prolapse. Laparoscopic procedures appear to be the better choice for POP treatment despite their higher cost, longer operative time and learning curve. Additionally, in laparoscopic procedures, the pelvic anatomy is better visualized, thus reducing the risk of intraoperative complications, promoting a quicker recovery, shortening the hospital stay and providing excellent cosmetic outcomes (16). At present, NOTES, including laparoendoscopic single-site surgery (LESS) and transvaginal natural orifice transluminal endoscopic surgery (vNOTES), is widely performed. Laparoscopic treatment includes uterine round ligament suspension, uterosacral ligament shortening, uterosacral ligament suspension (17), sacrospinous ligament fixation (SSLF), laparoscopic sacral colpopexy (LSCP) and pubic bone fixation. These surgical procedures shift from removing the bulging tissues and organs to strengthening the pelvic floor structure, thus maintaining the integrity of the pelvic floor support structure, with the advantages of rapid postoperative recovery and minimal trauma. In the current literature, researchers have identified laparoscopic sacral colpopexy (LSCP) as the gold standard treatment for apical prolapse, with success rates ranging from 78% to 100% (18). However, LSCP is a complex procedure requiring deep pelvic dissections and high-skilled suturing, in addition to being associated with rare but severe intraoperative complications (e.g., vascular injuries, sacral nerve root damage) (19, 20). Therefore, easier and less complex surgical reconstructive procedures that can guarantee the same anatomical and functional outcomes are needed. Currently, abdominal lateral suspension (LLS) with mesh is an alternative treatment that is performed to resuspend the vaginal apex and avoid possible damage caused by sacral promontory preparation or peritonization. The results from a systematic review suggest that LLS is safe, effective and feasible and produces optimal anatomical and functional outcomes (21). SSLF, a traditional surgery for apical prolapse, has good subjective and objective cure rates (5); however, by many patients experience postoperative complications. Gluteal pain is the most common complication of SSLF (7–12). In Mowat's study of POP patients who underwent SSLF with the Capio device, 86% reported buttock pain at 1 week, and the incidence had dropped to 16% by the sixth week (9). Nonsteroidal anti-inflammatory drugs (NSAIDs), local anesthesia, or surgical management are used to treat gluteal pain in some patients after SSLF (11–14). In Katrikh's study, the S4 nerve root was found in the medial third of the SSL in 96% (43/45) of cadaveric specimens. Sutures in this area during SSLF surgery may cause postoperative *de novo* perineal pain, urinary and fecal incontinence, and numbness of the genital and associated sacral root dermatome (22). The pudendal nerve and arteries are close to the ischial spine, and suturing at the lateral third of the SSL carries the risk of damaging these structures, possibly causing severe bleeding or nerve entrapment. Therefore, the placement of sutures in the middle segment of the SSL is less likely to damage the nerves or arteries (22). However, even when the middle third of the SSL is sutured, postoperative pain may still occur. In an anatomic study, innervation to the levator ani muscles and coccygeus arose from the S3 to S5 nerve roots, and 89% of the nerve branches to the levator ani muscles and/or coccygeus coursed over the mid-portion of the coccygeus-sacrospinous ligament (C-SSL) complex where SSLF sutures are usually placed (23). Another study also confirmed that the gluteal nerve was unlikely to be damaged during SSLF; however, branches from S3 and/or S4 perforated the ventral surface of the coccygeal muscles in 94% of specimens (6). Entrapment to those nerves may not only lead to buttock and posterior thigh pain but also lead to denervation of the pelvic floor muscles, thus increasing the risk of recurrent POP. Based on these results, Florian-Rodriguez suggested that careful dissection and exposure of the anterior surface of the C-SSL complex may allow visualization and avoid larger caliber nerve damage (6). The SSL is located deep in the pelvis, so its dissection and exposure during traditional transvaginal surgery are quite challenging. Some researchers have explored laparoscopic SSLF to ensure clear intraoperative exposure of the C-SSL complex and accurate suturing to it (24, 25); however, laparoscopic SSLF requires more extensive tissue dissection and a longer operative time, which increases the risk of severe intraoperative bleeding. In Kong's study, the mean operation time of laparoscopic SSLF was 117.78 ± 20.01 min, and the mean fixation time was approximately 30 min (24). In our study, single-port devices were placed when the right pararectal spaces were opened by sharp and blunt dissection, after which the connective tissue on the surface of the C-SSL complex was separated using an ultrasonic scalpel until the SSL was fully exposed. In this study, the operation time was 88.9 ± 10.2 min because less connective tissue needed to be laparoscopically dissected. More importantly, our procedures were performed in the extraperitoneal space, thus the absence of a pneumoperitoneum helped patients recover faster.

Life-threatening hemorrhage is another perioperative complication of SSLF, with reported occurrences ranging from 0.2% to 2% (26, 27). Directed compression and topical hemostatic agents can be used to control bleeding caused by vascular injury to the hypogastric and pudendal venous plexi (27). Arterial injuries, such as injuries to the inferior gluteal artery, require embolization (27). Barksdale et al. found an abundance of collateral blood supply and anastomosis with significant anatomical variation near the C-SSL complex, and the inferior gluteal artery and its coccyx branches are the most vulnerable to injury during surgery (28). Even though the suture is not placed in close proximity to the inferior gluteal artery (22), a blood vessel tear caused by separation or puncture during suturing can lead to life-threatening hemorrhage. In this study, no serious vascular damage occurred. When separation and suturing are performed laparoscopically, it is easy to locate and avoid blood vessels. The bipolar coagulator can be used to quickly and effectively stop bleeding if a blood vessel injury occurs during surgery.

Suturing the SSL is particularly difficult because the SSL is located deep in the pelvis and the surgical field of view is usually limited (29). In a cadaveric simulation model used to guide students in achieving optimal suture placement during SSLF, only 33% (3/9) of the students placed the sutures in anatomically safe locations during the first attempt (2.88 ± 2.10 attempts) (29). Out of 9 trainees in this study, 4 (44%) were previously able to perform SSLF independently, and among them, 3 (75%) did not succeed on the first try, and 2 required 6 attempts. It should be noted that in this study, if the trainee failed to sew the thread to SSL, it was routinely sutured to the iliococcygeal ligament (29), thus indicating that it is not easy for learners to master the skills of SSLF. As laparoscopy provides a clear field of view, it may be easier for learners to master.

Hysterectomy is usually performed to treat POP. However, studies have shown that uterine preservation in SSLF can reduce the operative time, risk of complications, and intraoperative blood loss without increasing the risk of POP recurrence (30, 31). Ng reported that after a mean follow-up of 13.3 years (range 8.5–22.6 years), there were no significant differences in the subjective success rate (89% vs. 88%) or current satisfaction (78.1% vs. 77.3%) rate between patients with and without uterus preservation during SSLF surgery (30). In Chou's study, the anatomical recurrence rate of POP was significantly lower in the uterine preservation SSLF group than in the concomitant hysterectomy group (11.5% vs. 45.5%, *P* = 0.039). Hysterectomy in SSLF was identified as a risk factor for anatomical recurrence (hazard ratio 4.08) (31). If there is no lesion in the cervix or uterus, we usually preserve the uterus, because it preserves the integrity of the sacral and main ligaments. In our study, the uterus was preserved in all the patients, and the cervical canal was sutured with nonabsorbable sutures. Because the cervix is a strong anchor point, the risk of suture avulsion is reduced. In addition, hysterectomy may destroy the integrity of the pelvic floor support structure. Studies have confirmed that sacrospinous ligament fixation with preservation of the uterus is not inferior to total transvaginal hysterectomy combined with sacrospinous ligament fixation for POP (32). For patients with cervical or uterine pathology (e.g., HISL) requiring hysterectomy, we will follow the steps in the literatures for removal of the uterus and management of the vessels on both sides of the uterus (33, 34). The vaginal stump is sutured and then the sub-Vnote sacrospinous ligament fixation is performed. Sutures fixed to the sacrospinous ligament are placed 1 cm below the posterior vaginal wall stump.

The operation has been performed for more than 60 years and has benefited a large number of prolapse patients. However, because the sacrospinous ligament is deep and difficult to expose, the operation is likely to cause damage to the peripheral blood vessels and nerves, and patients are likely to suffer from complications such as pain, bleeding, infection, and fistula. Because clinicians are discouraged from performing this operation, its global promotion has been greatly affected. In this work, we showed a new technique of transvaginal sacrospinous ligament fixation using single-port extraperitoneal laparoscopy for pelvic organ prolapse (POP) with narrated video footage (Supplementary Material), which well exposed the sacrospinous ligament. When the operation is performed under direct vision, the operator can completely avoid causing damage to the peripheral blood vessels and nerves. Improvements in this surgical method makes it easy to learn and master, thus increasing its global popularity. Because this is the first study of this procedure, we had a small sample of cases and no control group. Therefore, the effect of transvaginal single-port extraperitoneal laparoscopic SSLF should be confirmed in future clinical studies with large samples and long-term follow-ups.

## Conclusion

The results of our study indicate that transvaginal single-port extraperitoneal laparoscopic SSLF is a safe and effective operation for POP.

## Data Availability

The original contributions presented in the study are included in the article/**Supplementary material**, further inquiries can be directed to the corresponding authors.
